# Heterogeneity of Radiological Spectrum in Tacrolimus-Associated Encephalopathy after Lung Transplantation

**DOI:** 10.1155/2014/931808

**Published:** 2014-05-27

**Authors:** Qisi Wu, Christian Marescaux, Xinyue Qin, Romain Kessler, Jun Yang

**Affiliations:** ^1^Department of Neurology, The First Affiliated Hospital of Chongqing Medical University, 1 Youyi Road, Chongqing 400016, China; ^2^Stroke Unit and Department of Vascular Neurology, University Hospital of Strasbourg, 67000 Strasbourg, France; ^3^Department of Pulmonology, University Hospital of Strasbourg, 67000 Strasbourg, France

## Abstract

*Background*. Tacrolimus-associated encephalopathy (TAC-E) is usually described under the term of posterior reversible encephalopathy syndrome (PRES). However, a large amount of data has suggested that TAC-E is not a homogenous entity: indeed, TAC-E which is often presented with atypical and potentially misleading imaging characteristics does not always correspond to PRES. *Objective*. We aimed to identify the spectrum of brain MR imaging of TAC-E and discuss the underlying pathophysiological features. *Methods*. From September 2008 to October 2010, the neurological statuses of 45 patients, who underwent lung transplantation with TAC as posttransplantation immunosuppressive therapy, were regularly assessed in a prospective study. MRI was repeatedly performed, until recovery, in patients who developed central neurological symptoms. *Results*. Symptoms suggestive of encephalopathy occurred in five out of 45 patients (11.1%). According to our MRI study, two patients presented with reversible bilateral and relatively symmetric subcortical white matter edema with proximal vasospasms on MRA; however, three other patients were characterized by coexistence of two different lesions including laminar cortical infarcts with hemorrhagic transformation not typically found in PRES and reversible deep white matter edema, associated with distal vasospasms on MRA. *Conclusions*. It is considered that the mechanism of TAC-E would be more heterogenous than commonly perceived.

## 1. Introduction

Tacrolimus (TAC), one of calcineurin inhibitors (CNIs), revolutionized posttransplantation immunosuppressive therapy in the 1980s. This drug has been proven highly effective in preventing acute rejection. However, neurotoxicity, one of its major adverse effects, has been well documented particularly in solid organ transplantation (SOT) recipients [1–3]. TAC-associated encephalopathy (TAC-E), which is usually described under the term of posterior reversible encephalopathy syndrome (PRES), is considered as the most severe consequence. The term PRES that was initially identified by Hinchey et al. in 1996 described a potentially reversible imaging appearance and symptomatology that is shared by a diverse array of causes [4–9]. However, a large amount of data has suggested that TAC-E is not a homogenous entity: indeed, TAC-E which is not always reversible and is often presented with atypical and potentially misleading imaging characteristics does not always correspond to PRES, of which the distinctive magnetic resonance imaging (MRI) features were vasogenic/cytotoxic edema with frequently multifocal involvement from posterior to anterior regions. Therefore, from September 2008 to October 2010, we followed prospectively 45 patients, who underwent lung transplantation and under TAC as immunosuppressive therapy. Their neurological statuses were assessed regularly during this period. MRI was repeatedly performed, until recovery, in patients who developed severe central neurological symptoms. The goals of this prospective study were twofold (i) to describe the spectrum of the brain MRI findings in patients with TAC-E and (ii) to discuss the implications of these findings with regard to the pathophysiological features of TAC-E as well as the protocol for correctly identifying this entity so as to prevent deleterious workups or therapies.

## 2. Methods

From September 2008 to October 2010, 45 lung transplantations were performed in the University Hospital of Strasbourg, France, with posttransplantation immunosuppressive therapy of TAC. No other medication potentially causing PRES was recorded to be given to these patients. Patients who developed severe TAC-induced central neurological symptoms (visual impairment, seizures, confusion, or coma) were evaluated with repeated MRI examinations. TAC levels (recommended therapeutic range 5–15  **μ**g/mL) were regularly measured of which the highest level observed was recorded during neurological events. The time from initiation of TAC to onset of symptoms was recorded, too. Patients with only tremor, neuralgia, peripheral neuropathy, and brief episodes of visual hallucination were considered as having mild or moderate neurological symptoms and were excluded from this study.

Initial MRI was performed during the acute phase (within 24 hours) or subacute phase (1 day–3 weeks) in each neurological episode and follow-up MRI studies were repeatedly done until recovery and were reperformed one year later if possible. Fluid-attenuated inversion recovery sequences (FLAIR), diffusion-weighted magnetic resonance imaging (DWI), apparent diffusion coefficient (ADC), and T2∗-weighted imaging as well as 3D TOF MRA were included in our MRI protocol. All MRI studies were performed using a 3-T MRI (Philips). Diffusion-weighted imaging was performed in the axial projection with 3099/49 (TR/TE), 4-mm section thickness, and high-strength diffusion gradient (B: 1000 seconds/mm^2^) for acquisition times of 40 seconds per study. The MR images were interpreted separately by one radiologist and two neurologists, and final decisions on the findings were reached by a consensus. The observers assessed the presence, extent, and distribution of abnormal areas, signal intensity characteristics.

## 3. Results

### 3.1. Clinical Findings

Over the 2-year period of this study, we identified 9 clinical episodes in 5 patients (11.1%).

Seizures occurred in 6 episodes, of which there was focal onset in three and generalized seizures in three. Altered mental status was observed in 7 episodes and visual impairment in two. On clinical evaluation, the mean time from initiation of therapy to the onset of clinical presentations was 12 days (range: 3–27 days). TAC level data were available for 8 episodes and demonstrated a mean TAC level of 20.0 (range: 4.6–28.6 **μ**g/L), which was higher than its therapeutic range.  Table 1 provided the clinical characteristics regarding these patients.

### 3.2. Radiological Findings

In all 9 episodes initial neuroimaging with MRI of the head was done. The most commonly involved location was the parietooccipital brain region, which was seen in all 9 episodes. This was followed by the frontal lobe in 6 episodes, the deep white matter in four, the cerebellum in three, the basal ganglia in two, the brainstem in two, and the thalamus and temporal lobe in one, respectively. Of the episodes, seven had subcortical involvement and five had cortical involvement. Lesions were unilateral in 1 episode. Hemorrhage was present in 5 episodes. Contrast enhancement was evident in two.

**Figure 1:**
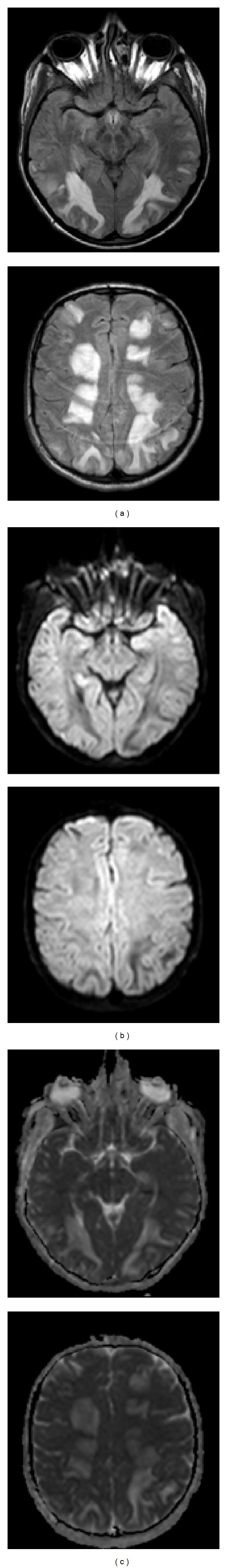
MRI manifestations of Patient 1. (a) Axial fluid attenuated inversion recovery (FLAIR) showed symmetrical high signal intensities in subcortical occipito-parietofrontal regions. ((b)-(c)) Diffusion-weighted magnetic resonance imaging (DWI) revealed hypointensities with high apparent diffusion coefficient (ADC), suggesting vasogenic edema.

**Figure 2:**
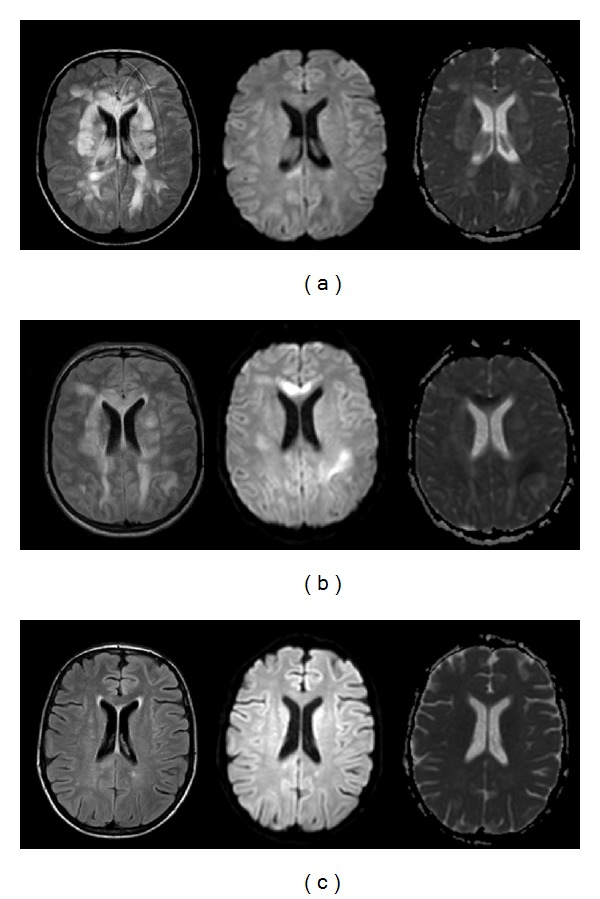
MRI evolutions in Patient 1. (a) Deep white matter vasogenic edema. (b) Incomplete regression of vasogenic edema with cytotoxic edema in corpus callosum and parietal regions. (c) A remarkable resolution of vasogenic/cytotoxic edema.

**Figure 3:**
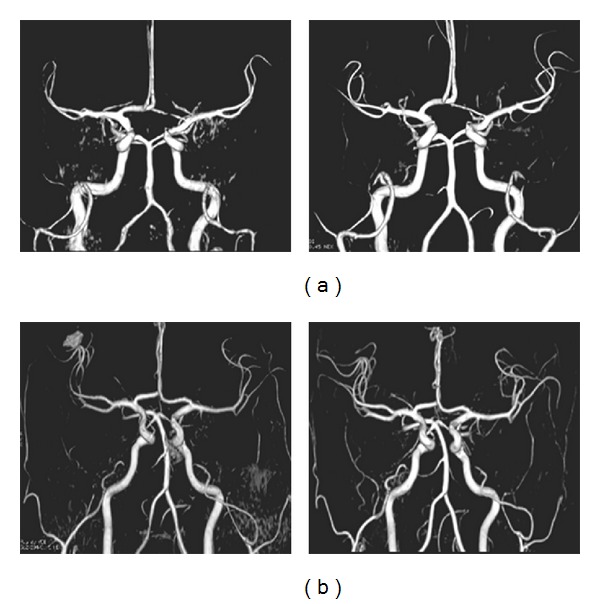
MRA evolutions in Patient 1 and Patient 3. (a) Reversible vasospasm of vertebral arteries with PCA, MCA, and ACA spasms in Patient 1. (b) Reversible vasospasm of vertebrobasilar arteries along with invisibility of more distal cerebral arteries in Patient 3.

When analyzed according to clinical subgroups, imaging characteristics, including lesion distribution and type, were varied. Two patients underwent lung transplantation due to mucoviscidosis (Patients 1 and 2; age range: 14–19 years, mean age: 16.5 years). Both of them had no cortical involvement but brain edema with hyperintense signal in FLAIR and ADC, suggesting vasogenic edema, located in not only the occipito-parietofrontal lobes (Figure 1) but also in deep white matter, basal ganglia, thalami, cerebellum, and brain stem (Figure 2(a)). Relative symmetry was seen in both patients. One patient (Patient 1) developed additional lesions with decreased ADC values, demonstrating cytotoxic edema (Figure 2(b)), involving frontoparietal lobe as well as corpus callosum and thalamus. Additionally, in both patients, diffuse proximal vasospasm appeared on MRA including vertebral and basal artery, middle cerebral artery (MCA), anterior cerebral artery (ACA), and posterior cerebral artery (PCA) (Figure 3(a)). However, on follow-up, remarkable resolution of edema and vasospasmwas revealed after the withdrawal of TAC (Figures 2(c) and 3(a)).

Two patients underwent lung transplantation due to chronic obstructive pulmonary disease (COPD) and one due to silicosis (Patients 3, 4, and 5; age range: 47–60 years, mean age: 54.3 years).

**Figure 4:**
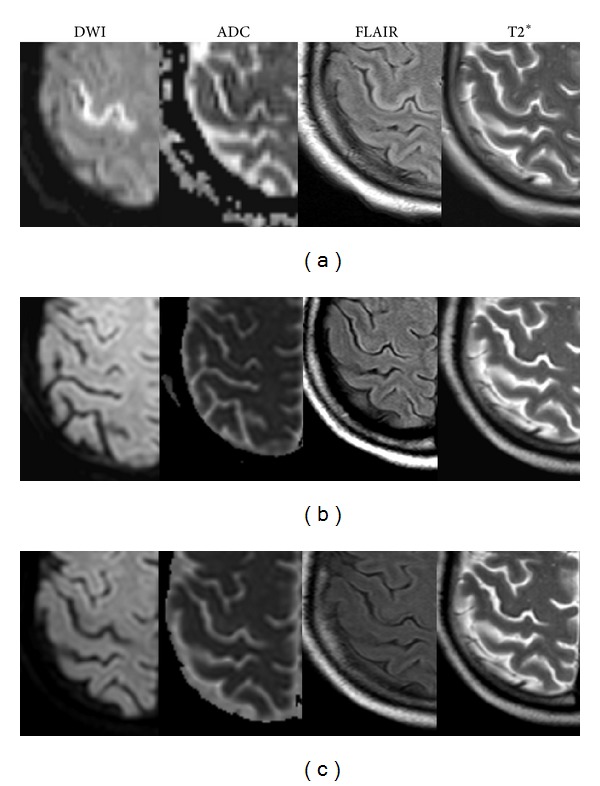
Laminar cortical infarcts with hemorrhagic transformation. (a) T2∗ showed asymmetrical hyperintense cortical lesions in right Rolandic region with restricted diffusion. (b) T2∗ showed hypointense signal changes in the same region, suggestive of hemorrhagic transformation. (c) No resolution of cortical lesions.

**Figure 5:**
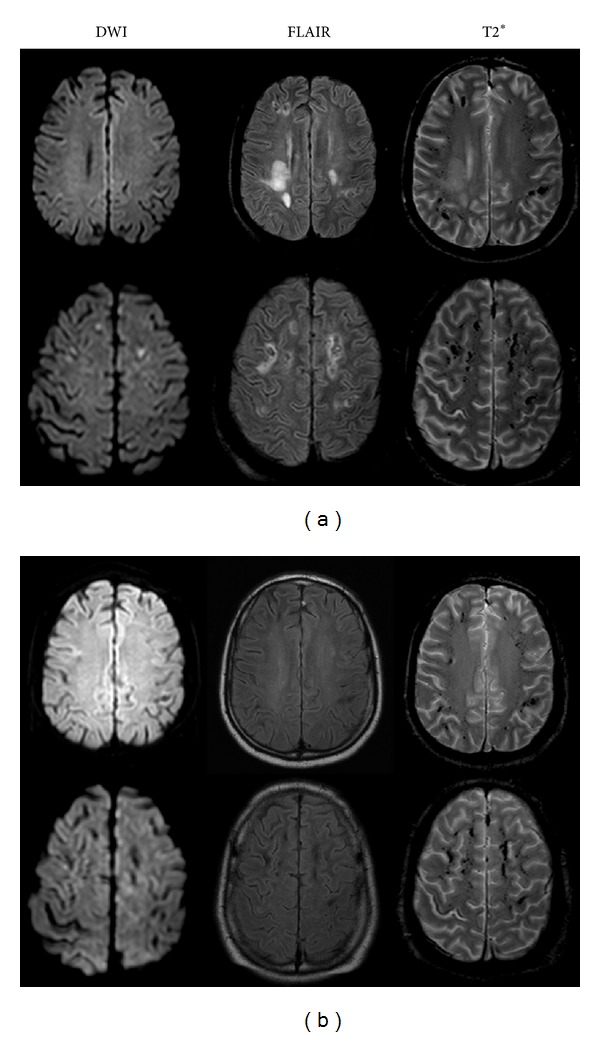
MRI manifestations of Patient 3. (a) Deep white matter edema in frontoparietal lobe with hemorrhagic lesions. (b) Resolution of brain edema with residual hemorrhagic lesions.

All three patients presented with multifocal cortical involvement located with laminar distributions in the cortex of occipital lobe, Rolandic region, and frontoparietal lobe with hyperintensity signal change on DWI with decreased ADC values, along with a slight thickening and hyperintensity of the pre-Rolandic cortex on FLAIR, showing evidence of infarct on initial MRI (Figure 4(a)). Meanwhile, contrast-enhanced T1-weighted images showing cortical enhancement were seen in two. However, within three weeks (range: 6 days–3 weeks), the follow-up T2 or T2∗-weighted images showed hypointense changes in the same regions (Figure 4(b)), suggestive of hemorrhagic transformation. The lesions were therefore interpreted as laminar cortical infarcts with hemorrhagic transformation. Besides, on initial MRI studies, additional deep white matter vasogenic/cytotoxic edema (Figure 5) was found in all three patients in occipital lobe, frontoparietal lobe, and cerebellum, respectively. Asymmetry was seen in all of them including 1 with unilateral distribution. Besides, 1 patient demonstrated dot-like hemorrhage that also could be seen in atypical PRES. Furthermore, distal vasospasms were observed on MRA (Figure 3(b)). Except the dot-like hemorrhage and cortical lesions which showed a hemorrhagic transformation, all the other presentations including brain edema on MRI and vasospasm on MRA were reversible after the withdrawal of TAC at the follow-up studies (Figures 3(b), 4(c), and 5(b)).

## 4. Discussion

TAC-induced neurotoxicity has been well documented particularly in SOT recipients. As both sensorial motoric functions may be adversely affected, patients thus present with a wide range of neurological and psychiatrical disorders. Mild and moderate symptoms include tremor, neuralgia, peripheral neuropathy, and transient visual hallucinations. Severe symptoms are manifested as seizures, confusion, coma, and visual impairment [2, 3, 10–16]. It was reported that the major side effects of TAC occurred in 5% to 8% of transplant recipients and the incidence of TAC-induced PRES was approximately 1.6% [10, 17]. Our study indicated that the incidence of TAC-E was 11.1%, which was higher than that suggested by older reports or studies. Moreover, as in our study, we excluded patients with only presentation of brief visual hallucinations which is considered as mild or moderate symptom, and some TAC-E patient could have been missed as TAC-E symptoms sometimes are slight and transient and thus potentially have underscored the frequency of TAC-E in this study.

## 5. Neuroimaging Features

In our study, two patients (Patients 1 and 2) presented with reversible bilateral and relatively symmetric subcortical white matter edema, associated with proximal vasospasms on MRA; however, three other patients (Patients 3, 4, and 5) were characterized by coexistence of two different lesions including reversible deep white matter edema and laminar cortical infarcts with hemorrhagic transformation, associated with distal vasospasms on MRA. We are therefore suggesting that the manifestations of TAC-E would be more heterogenous than commonly perceived, and MRI is essential to diagnose TAC-E. FLAIR is the most sensitive sequence for recognition of edema [5]. Yet new MR techniques, including DWI and ADC, reliably distinguish not only vasogenic/cytotoxic edema [8, 18, 19] (Table 2) but also laminar cortical infarct as DW imaging has proved to be an accurate and a relatively quick method for early diagnosis of both focal and global cerebral infarct with hyperintensity on DW images resulting from cytotoxic edema and restricted diffusion of free water [20, 21].

Neuroimaging features of TAC-E are classically associated with bilateral and symmetric brain edema in subcortical regions of the parietal and occipital lobes, corresponding to typical manifestation of PRES, in which vasogenic edema rather than cytotoxic edema may play a pivotal role in neuroimaging characteristics [7, 8, 19, 22], which could be seen in Patients 1 and 2. In most of our patients, however, lesions were rarely limited to the posterior regions of the brain and asymmetric involvement of at least one brain region or even a unilateral variant was observed. Posterior frontal lobe, temporal lobe, cerebellum, thalamus, brainstem, deep white matter, and basal ganglia which used to be called atypical PRES presentations but with a higher incidence than commonly perceived were all affected in our patients.

Additionally, special attention was given to irreversible cortical abnormalities with hemorrhagic transformation in three other patients. According to initial MRI, lesions located in the cortex showed hyperintensity signal change on DWI with decreased ADC values with contrast-enhanced T1-weighted images showing cortical enhancement, suggesting severe ischemic injury due to low perfusion [20]. Yet, as our patients were not imaged in hyperacute phase after ictus (within 30 minutes), the abnormal signal changes on DWI and ADC may be more related to infarction rather than ischemia [21]. The cortical infarcts could probably result in cortical necrosis (“laminar cortical necrosis,” reflecting pannecrosis, that is, the death of neurons, glia, and blood vessels in the affected area, resulting in protein degradation), which has already been reported in the patients with TAC-induced neurotoxicity [10, 23]. However, on follow-up visits, hypointense changes on T2∗-weighted images developed in the same regions without cortical atrophy, which was rarely found in the progression of laminar cortical necrosis [24–26]. And in our patients, white matter lesions occurred at the same time of the cortical abnormalities, which were reversible in the later stage. Both findings were not consistent with the findings of laminar cortical necrosis that in the chronic stage cortical atrophy and delayed but progressive white matter changes were seen [20, 27]. In addition, T1-weighted imaging has been recommended to show possible laminar necrosis. On T1-weighted imaging, high-signal cortical lesions were reported to be found 2 weeks after the ictus, which became prominent at 1–3 months and then less obvious [28]. However, we did not observe these chronological changes on T1 in our patients. Therefore, in our study, the MRI manifestations did not correspond to typical presentation of laminar cortical necrosis, and laminar cortical infarcts could be more appropriate to explain the cortical lesions in our patients.

Moreover, in our study, the follow-up T2∗-weighted images showed hypointense changes in the same regions of cortical infarcts. T2∗-weighted magnetic resonance imaging as well as susceptibility-weighted imaging (SWI) is capable of showing not only haemorrhage but also mineral deposition (e.g., iron, calcium), all seen as hypointensities [29]. And similar cortical T2∗ hypointensity changes, which were interpreted as an iron and/or calcium deposition rather than a hemorrhagic process, were reported in seizures or mitochondrial encephalopathy, lactic acidosis, and stroke-like episodes (MELAS) patients [29–31]. Yet, in our case, the patients with atypical laminar cortical infarcts did not seem to have more often seizures than the patients without cortical infarcts. As a result, the T2∗ abnormalities were interpreted as hemorrhagic transformation rather than mineral deposition in our series. Thus, we are in favor of “laminar cortical infarcts with hemorrhagic transformation” as the possible mechanism of laminar cortical abnormalities in our patients.

## 6. Pathophysiology

It is known that neurological alterations after CNI therapy are associated with brain structures showing high calcineurin expression [32]. But the precise mechanism of TAC-E is still incompletely understood. From a historical perspective, two major hypotheses to explain the MRI presentations of PRES were discussed in the literature, hyperperfusion and hypoperfusion theory. The most popular hypothesis is related to hypertensive encephalopathy [33]. Severe hypertension leads to transient disruption of autoregulation system consisting of a myogenic and a neurogenic response (sympathetic innervation) which will lead to cerebral vasodilatation, and hence allowing extravasation of fluid and blood into the brain parenchyma causing vasogenic cerebral edema [34]. However, theories regarding the pathophysiology of TAC-induced hypertension remain controversial, and the majority of the patients reported in the literature had no history of high blood pressure at clinical onset.

Alternatively, another hypothesis is the arterial endothelial injury by toxic drug effect [35]. Experimental data has indicated direct cytotoxic effects on the brain capillary endothelial cells with TAC [36]. And it is suggested that with release of vasoactive substances such as endothelin due to disrupt endothelial integrity, spasm in cerebral arteries would be likely seen in neurotoxicity induced by TAC [10, 37]. Endothelin could gain access to the cerebral vascular smooth muscle, resulting in vasoconstriction and vasospasm. Elevated circulating endothelin could also promote systemic hypertension. Under such conditions, some of the neuroradiologic findings could show typical transient white matter edema in the subcortical parietal and occipital lobes, as observed in cases of acute hypertensive encephalopathy. As in our two patients with typical TAC-E (Patients 1 and 2), elevated blood pressure and TAC levels were observed in most episodes. And the imaging studies have demonstrated vasogenic/cytotoxic edema in subcortical regions of parietal and occipital lobes along with diffuse proximal vasospasm resulting in hypoperfusion. We are therefore suggesting that TAC-induced vasculopathy associated with proximal vasospasm leading to brain hypoperfusion and presumed ischemia would be more appropriate mechanism of typical TAC-E.

Furthermore, as laminar cortical infarcts with hemorrhagic transformation were revealed in our study, other unique pathophysiological considerations were implied. As it was described, increased release of endothelin which was related to TAC-induced neurotoxicity could result in vasoconstriction and vasospasm. If the vasospasm is prolonged, a hypoxic episode could result and lesion would be similar to that described in hypoxic encephalopathy [10]. In our three patients with atypical cortical abnormalities, the evolution of findings on the imaging studies indicated that they probably suffered a hypoxic episode. According to initial MRI of these patients, lesions located in the cortex showed hyperintensity signal change on DWI with decreased ADC values indicating cortical infarction that could also be found in patients with hypoxic encephalopathy [21], with contrast-enhanced T1-weighted images showing cortical enhancement which demonstrated blood-brain barrier (BBB) disturbance. An increase in the permeability of the BBB can facilitate the passage of TAC, thus promoting cerebral ischemia and extending neurologic damage. And the development of cortical laminar infarcts may indicate that a hypoxic or hypoperfusion episode has occurred due to prolonged distal vasospasm that was also seen in our three patients. Moreover, hemorrhagic transformation after cortical infarcts which was rarely seen in PRES was demonstrated in our study. It might be related to the disruption of the BBB along with the disturbances of local cortical microvascularization attributable to vasoconstriction of small arteries and arterioles, which was similar to pathophysiology of hemorrhagic transformation of acute ischemic stroke [38]. In addition, it was noticed that the three patients with laminar cortical infarcts along with hemorrhagic transformation were also clearly older (mean age: 54.3 years) than the two patients with typical TAC-E (mean age: 16.5 years), which indicated that age may play a role in the occurrence of hemorrhagical lesions in TAC-E. Further work is needed. Thus, it was considered that the possible pathophysiological mechanism of atypical MRI manifestation could be vasculopathy along with a possibly more severe, prolonged, or distal vasospasm leading to laminar cortical infarcts with hemorrhagic transformation, which would result in the irreversibility of the cortical lesions.

## 7. Conclusions

It is considered that the mechanism of TAC-E would be more heterogenous than commonly perceived. However, Lee et al. [39] suggested that although there are a variety of superficially unrelated precipitants involved in the process, all the triggers are presumably unified by a final common pathway, possibly dysfunction of cerebral autoregulation.

## Figures and Tables

**Table 1:** Clinical characteristics of 9 episodes of TAC-E of the 5 patients.

Patient number/episode number	Age (y)/sex	Comorbidities	Clinical symptoms	Highest TAC level (ug/L)
1/1	19/F	Mucoviscidosis, BPHT	Headache, generalized tonic-clonic seizures, and altered mental status	11.7
1/2	19/F	Mucoviscidosis, BPHT	Confusion, epileptoid trepidation on right foot	28.6
1/3	19/F	Mucoviscidosis, BPHT	Convulsions, coma	4.6
2/4	14/F	Mucoviscidose BPT	Headache, diplopie, and blurred vision	28.2
3/5	47/M	Silicosis, BPT	Myoclonus	20.8
3/6	47/M	Silicosis, BPT	Vomiting and apathy, confusion	18.8
3/7	47/M	Silicosis, BPT	Myoclonus, coma	NA
4/8	56/F	COPD, BPT	Generalized tonic-clonic seizures with right hemiplegia, confusion	25.1
5/9	60/M	COPD, BPT	Visual and auditory hallucinations, confusion	22.5

COPD: chronic obstructive pulmonary disease; NA: not available; BPHT: bipulmonary and hepatic transplantation; BPT: bipulmonary transplantation; CPT: cardiopulmonary transplantation.

**Table 2:** MRI alternations of vasogenic and cytotoxic edema in TAC-E.

	Vasogenic edema			Cytotoxic edema
FLAIR (T2-weighted or proton density weighted images)	*↗*	*↗*	*↗*	*↗*
DWI	N	*↗*	*↘*	*↗*
ADC	*↗*	*↗*	*↗*	*↘*
Evolution	Usually reversible			Usually irreversible or can be reversible partially or totally

*↗* indicates hyperintense signal; N: isointense signal; *↘*: hypointense signal.
